# Highly Active and Durable Nanostructured Nickel‐Molybdenum Coatings as Hydrogen Electrocatalysts via Solution Precursor Plasma Spraying

**DOI:** 10.1002/open.202400069

**Published:** 2024-10-25

**Authors:** Xiuyu Wu, Alexis Piñeiro‐García, Mouna Rafei, Alice Kuzhikandathil, Esdras J. Canto‐Aguilar, Eduardo Gracia‐Espino

**Affiliations:** ^1^ Department of Physics Umeå University SE-901 87 Umeå Sweden; ^2^ Departamento de Ingeniería Química Alimentos y Ambiental Universidad de las Américas Puebla Sta. Catarina Mártir, Cholula, Puebla 72810 Mexico

**Keywords:** Hydrogen, Nickel-molybdenum, Electrochemistry, Plasma spraying

## Abstract

The increasing demand for green hydrogen is driving the development of efficient and durable electrocatalysts for the hydrogen evolution reaction (HER). Nickel‐molybdenum (NiMo) alloys are among the best HER electrocatalysts in alkaline electrolytes, and here we report a scalable solution precursor plasma spraying (SPPS) process to produce the highly active Ni_4_Mo electrocatalysts directly onto metallic substrates. The NiMo coating coated onto inexpensive Ni mesh revealed an excellent HER performance with an overpotential of only 26 mV at −10 mA cm^−2^ with a Tafel slope of 55 mV dec^−1^. Excellent operational stability with minimum changes in overpotential were also observed even after extensive 60 hour high‐current stability test. In addition, we investigate the influence of different substrates over the catalytic performance and operational stability. We also proposed that a slow, but consistent, dissolution of Mo is the primary degradation mechanism of NiMo‐based coatings. This unique SPPS approach enables the scalable production of exceptional NiMo electrocatalysts with remarkable activity and durability, positioning them as ideal cathode materials for practical applications in alkaline water electrolysers.

## Graphical abstract

Scalable solution precursor plasma spraying yields high‐activity, large‐surface‐area, and durable NiMo electrocatalysts for hydrogen production via alkaline water electrolysers. The influence of the substrate and Mo dissolution on the catalytic performance and operational stability is discussed.

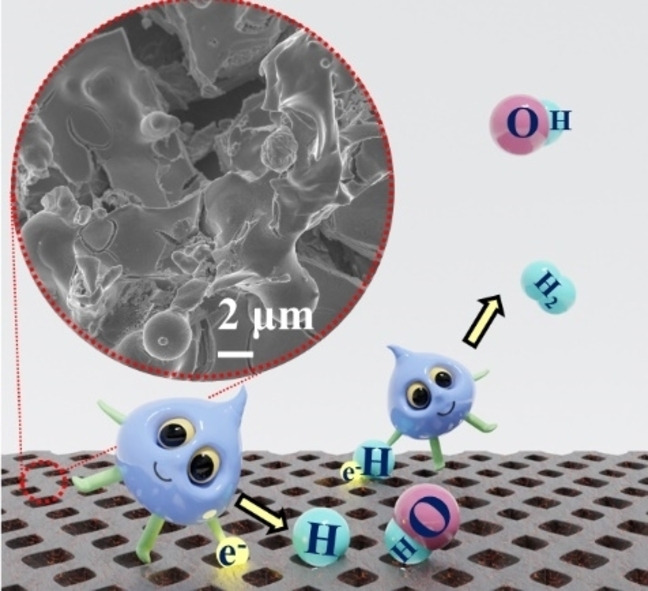

## Introduction

Green hydrogen, as a non‐toxic and clean energy carrier, holds immense potential for enabling a sustainable energy system with minimal greenhouse gas emissions, particularly CO_2_.^[1]^ In recent years, global demand for hydrogen has experienced a tremendous increase reaching 94 million tonnes (Mt) in 2021, and it is projected that nearly 200 Mt will be required by 2050 to meet the net zero CO_2_ emissions scenario.^[2]^ Currently, more than 96 % of the total hydrogen production relies on fossil fuels resulting in significant CO_2_ emissions and air pollution.^[3]^ Green hydrogen production is projected to be produced via alkaline water electrolysis technology due to better scalability, availability of non‐noble metal electrocatalysts, simpler designs, and longer lifespan when compared to other water electrolysis technologies.^[4]^

Platinum has been considered one of the best electrocatalyst for the HER in acid media. However, the slow water dissociation reaction required in alkaline water electrolysis, makes Ni‐based electrocatalysts a good alternative for Pt.^[5]^ Despite the high abundance and low cost of Ni, its poor HER activity and progressive deactivation, due to poisoning with surface‐adsorbed hydrogen by forming passive NiH_x_ layer,^[6]^ limits its use in practical applications. Nevertheless, the encouraging electrochemical performance Ni has motivated the design of various Ni alloys, where nickel‐molybdenum alloys are widely acknowledged as outstanding HER electrocatalysts.^[7]^

It has been reported that the presence of molybdenum in NiMo alloys results in improved hydrogen adsorption energies and water dissociation capabilities, facilitating the otherwise sluggish HER kinetics observed in Ni‐based materials.[^7^, ^8^] A large variety of synthesis methods have been employed to produce NiMo‐based electrocatalysts, such as electrodeposition,^[9]^ hydrothermal,[^9^, ^10^] mechanical alloying,^[11]^ atmospheric plasma spraying,[^7^, ^12^] and electrospinning.^[13]^ Although, the durability of these electrocatalysts has been found to be less than ideal with Mo corrosion observed in several cases.[^10^, ^14^] C. Li and collaborators reported a NiMo alloy coupled with Ni(OH)_2_ nanosheets that exhibited nearly 40 % of Mo dissolution leading to a reduced HER activity.^[10b]^ Similarly, Du, W. and coworkers produced the highly active Ni_4_Mo supported onto nickel foam, and after 4 h of operation nearly 40 % of the Mo was lost, while after 12 h of operation the current response was reduced by 50 % when the electrolyte was renewed every hour.^[14b]^

In this work, we report the production of nanostructured NiMo coatings using a fast and scalable SPPS process. From simple Ni−Mo water‐based solutions, we produce metallic NiMo coatings on Ni and stainless‐steel current collectors, which exhibit large electrochemical surface area, excellent activity towards the HER in alkaline, and great durability even at high current densities. We also observed a synergetic effect between the current collector and the active material, being Ni‐mesh the best material in terms of electrical conductivity and corrosion resistance, making it a promising electrode configuration to perform the HER in harsh alkaline conditions.

## Experimental Section

### Electrocatalytic Coatings

Ni(NO_3_)_2_ ⋅ 6H_2_O, and (NH_4_)_6_Mo_7_O_24_ ⋅ 4H_2_O (81–83 %) were purchased from Merck. The precursor solutions were prepared by dissolving the metal precursors in deionized (DI) water (Milli‐Q, 18.25 MΩ). The total metal concentration was fixed to 0.40 M with a Ni : Mo ratio of 5 : 2 (Mo=28.5 at.%). The electrocatalytic coatings were prepared via solution precursor plasma spraying (SPPS).^[15]^ An atmospheric plasma spraying equipment (Metallisation, Met‐PCC(PLAS)) with a PL50 pistol equipped and a 6 mm anode was used. Two metallic substrates were used to deposit the catalytic coating: (i) Stainless‐steel mesh (SS‐mesh, SS316, 45 μm sieve size), and (ii) diamond‐shaped expanded Ni mesh with a thickness of 0.76 mm, wire width of 0.88 mm, and a short and long way of mesh of 1 and 3 mm, respectively. Before the coating deposition, the SS‐mesh was cleaned using an ultrasonic bath in a mixture of DI water and ethanol (50 : 50 v/v) for 30 min following by drying with compressed air. The Ni mesh was cleaned by performing a mild acid pickling using 20 % HCl (aq.) at 60 °C for 1 min, then rinsed with DI water for several times. A homogeneous coating was formed using a plasma flame generated with a mixture of Ar (50 NL min^−1^) and N_2_ (2.0 NL min^−1^) gases while applying a direct current of 500 A. The spraying distance from the plasma torch to the substrate was fixed at 20 cm. The precursor solution (15 mL min^−1^) was premixed with N_2_ gas (3 NL min^−1^) before injection into the plasma plume using a high‐pressure syringe pump. The plasma torch was manipulated by a robotic arm (ABB 2600) following a raster scan pattern as shown in Figure S1. The lateral velocity of the torch was 250 mm s^−1^ with a vertical displacement of 4 mm covering an area of 7 cm ×5 cm. The coating was formed by applying 5 layers, only one side for SS‐mesh, but both sides on Ni‐mesh. Afterwards, the as‐sprayed substrates were annealed in Ar:H_2_ (95 : 5) atmosphere at 450 °C for 2 hours (temperature ramp 22.5 °C min^−1^). The samples were cooled down in the same atmosphere and stored for further analysis.

### Material Characterization

X‐ray diffraction (XRD) patterns were measured on a PANalytical X'pert powder diffractometer equipped with Cu K_α_ radiation (λ=1.5406 Å) in the range of 5–80° at ambient temperature. A step size and time per step of 0.01395° and 0.5 s were used, respectively. Scanning electron microscopy (SEM) studies were carried out on Carl Zeiss Merlin equipped with energy dispersive X‐ray spectroscopy (EDS). High‐resolution transmission electron microscopy (HRTEM) studies were performed on a FEI Titan Krios. The samples were prepared by collecting the sprayed material from a glass substrate after removing it with a ceramic blade. The collected powder was dispersed in ethanol and drop‐casted onto a TEM grid. X‐ray photoelectron spectroscopy (XPS) analyses were conducted on an Axis Ultra DLD electron spectrometer (Kratos Analytical) equipped with a monochromatic X‐ray source (Al K line of 1486.6 eV). The spectra were charge corrected to C 1s set to 284.3 eV (C−C sp^2^). Raman spectra were recorded in a Renishaw Qontor Raman spectrometer using a 532 nm laser diode calibrated with a Si crystal at 521 cm^−1^, the laser was operated at 5 % of power during 3 s with a total of 3 accumulations.

### Electrochemical Characterization

The electrochemical measurements were performed using a potentiostat (Ivium Technologies) in a three‐electrode cell containing Ar‐saturated 1 M KOH as electrolyte at room temperature. The reference and counter electrodes were Ag/AgCl (70 mm, 3 M KCl) and a graphite rod, respectively. The working electrodes were catalytic coatings on SS‐mesh, or Ni‐mesh with an exposed geometric area of 1 cm^2^. The activity of the electrodes was evaluated using cyclic voltammetry (CV) at a scan rate of 5 mV s^−1^ in the range of −1.036 to −1.436 V vs. Ag/AgCl. A total of 9 CV scans were carried out to achieve a stable behaviour, and the last one is reported in this work. The measured potential was converted to the reversible hydrogen electrode (RHE) potential using E_vs.RHE_=E_vs.Ag/AgCl_ +0.210+0.059×pH ‐ 0.95iR_Ω_. The ohmic resistance R_Ω_ of the electrochemical system was measured by electrochemical impedance spectroscopy (EIS) at potential required to achieve a current density of −10 mA cm^−2^ in a frequency range of 0.1 Hz to 10 kHz with an amplitude of 10 mV. The operational stability was evaluated with 5000 CV cycles (0.05 to −0.15 V vs. RHE) at a scan rate of 50 mV s^−1^, followed by a chronoamperometric (CA) test at a potential required to achieve −50 mA cm^−2^ for 48 h. The double layer capacitance was evaluated by conducting several CVs in a non‐Faradaic region at different scan rates (5, 20, 40, 60, 80 and 100 mV s^−1^).

## Results and Discussion

### Characteristics of the NiMo Coating

**Figure 1:**
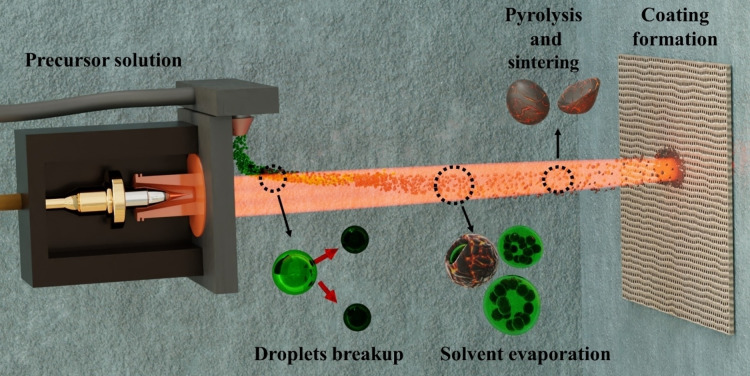
Schematic representation of the coating process onto a given substrate via solution precursor plasma spraying using a solution containing the desired metal precursors. The precursor solution experience droplet breakup, solvent evaporation, pyrolysis, and sintering in‐flight. The final coating exhibit nanostructured features.

Plasma spraying in form of SPPS in an excellent technique to produce nanostructured homogenous coatings containing several transition metals.^[15]^ During our SPPS process, an aqueous solution containing the metal precursors (Ni−Mo) is injected perpendicularly to the plasma plume, as depicted in Figure 1. Inside the plasma, the drops breakup into finer droplets due to their different velocity relative to the plasma flow. The droplets are then heated up rapidly causing solvent evaporation.^[16]^ Finer droplets that find its way to the plasma core undergoes precursor precipitation, pyrolysis, and sintering (particle formation). The resulting solid particles are melted and sputtered onto the substrate forming the coating. Meanwhile, droplets in low temperature areas in the plasma reach the substrate without experiencing the entire process, forming spherical hollowed particles.^[17]^ All these results in a variety of morphologies with nanostructured features which offer a large surface area, a desired characteristics in electrocatalysis.^[15]^ Finally, the coating is formed by a successive deposition of particles while moving the plasma torch in a raster scan pattern (**Figure** **S1**) until the desired loading has been achieved.

**Figure 2:**
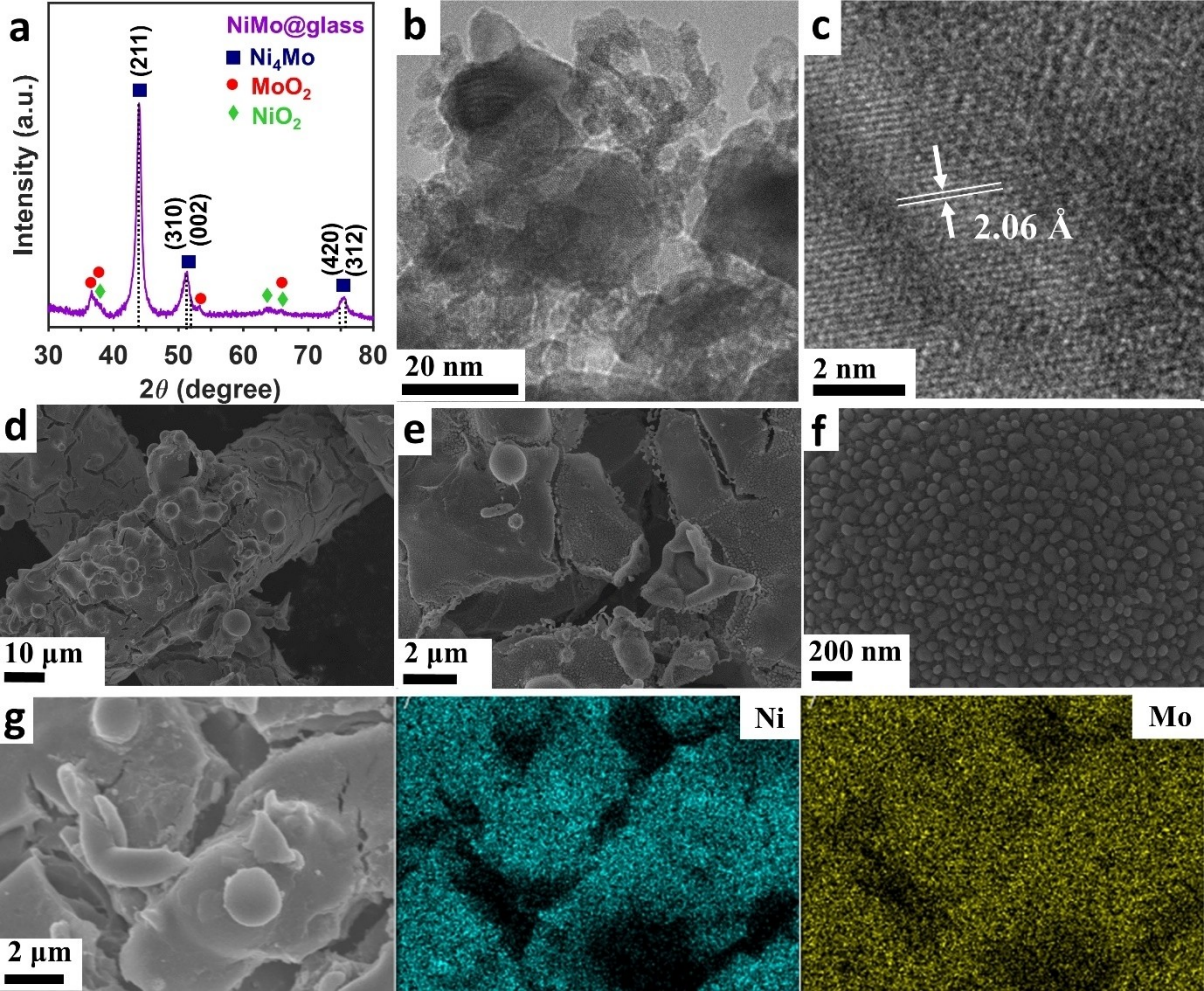
(a) X‐ray diffractogram and (b–c) HRTEM images of NiMo@glass. The arrows in (c) indicate the average interplanar distance. (d‐f) SEM images and (g) EDX elemental mapping of NiMo@SS.

The NiMo coating was grown onto three different substrates: (i) stainless‐steel mesh (SS‐mesh), (ii) nickel mesh (Ni‐mesh), and (iii) glass slide. Further details can be found in the experimental section. The coating sprayed on glass was used for X‐ray powder diffraction (XRD) studies to avoid features arising from both SS‐mesh and Ni‐mesh substrates that overlap with those corresponding to the coating (see Figure S2). Note that NiMo@glass was only used to identify the XRD features of the coating, as the substrate might have effects on finer structural details such as porosity, surface area, splat characteristics, and coating adhesion that might affect the catalytic performance. The XRD patten of NiMo@glass can be seen in Figure 2a. It is clear that the coating comprises the tetragonal Ni_4_Mo (materials project, mp‐11507) with features at 43.9° corresponding to the (211) plane, 51.3° to both (310) and (002), and 75.5° to (420) and (312) crystal planes.^[18]^ An average crystalline size of 9.5 nm was obtained when using the Debye‐Scherrer equation (β=0.9) in the (211) peak. XRD studies also shows the potential contribution of metal oxides, such as NiO_2_ (mp‐25210) and/or monoclinic MoO_2_ (mp‐559140), likely formed due to the excess of both Ni and Mo in the precursor solution (Ni : Mo=5 : 2). The microstructure of NiMo was further characterized by high‐resolution TEM (Figure 2b) where well‐defined crystalline nanoparticles are clearly observed, with some depicting an average interplanar distance of 2.06 Å in agreement with the (211) plane of Ni_4_Mo (Figure 2c).

The surface morphologies were examined by SEM studies, the corresponding images are depicted in Figures 2d–f and S3 for NiMo@SS, and Figure S4a–d for NiMo@Ni. Both substrates are entirely coated with no visible exposed areas. The coating comprises micrometre size (1–10 μm) agglomerates in form of spheres, hollow spheres, and flakes. These agglomerates are composed of polycrystalline nanoparticles, as evidenced by HRTEM and XRD, such characteristics are commonly observed in coatings produced by SPPS.[^15^, ^19^] The formation of these microstructures occur during the coating process before the thermal annealing, see Figure S5, in agreement with previous studies where particles formation occurs in‐flight during the spraying process.^[17]^ After the annealing process there is a reduction in the oxygen content (Figure S5c–d) and the appearance of superficial fissures (Figure 2d–e and Figures S3–S5). The fissures could have being originated due to variations of expansion coefficients between the coating and substrate, as well as remnants of solvent or precursor decomposition.^[20]^ A significant difference seen between the coatings was the average size of the nanostructured particles, where NiMo@SS and NiMo@Ni exhibit nanoparticles of ~60 nm and ~20 nm in size, respectively (see Figure S6). Indicating that Ni as substrate notably decreases the particle size.

Furthermore, EDX elemental mapping (Figure 2g) shows a homogeneous distribution of Ni and Mo with no clear signs of segregation, also indicating a uniform distribution and the nano‐size nature of all the crystalline phases composing the micrometric particles deposited on the substrates. Note that the black areas in the EDX maps correspond to differences in the surface topography containing low‐altitude areas (from fissures) and high‐altitude areas from agglomerates. The obtained Ni : Mo atomic ratio was 5 : 1.8 for NiMo@SS and 5 : 2.3 for NiMo@Ni (see Table S1), which is consistent with the composition of the solution precursor (Ni : Mo 5 : 2).

### Surface Chemical States of NiMo

**Figure 3:**
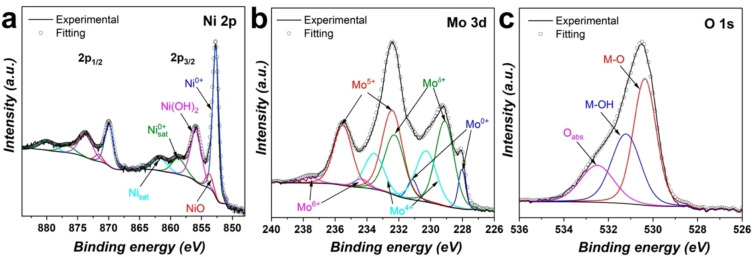
High‐resolution XPS spectra of Ni 2p (a), Mo 3d (b), and O 1s (c) of NiMo@SS.

The surface chemical compositions and oxidation states of NiMo@SS electrodes were analysed by X‐ray photoelectron spectroscopy (XPS). XPS survey spectrum (Figure S7) confirms the presence of Ni and Mo on the electrode surface. The high‐resolution Ni 2p spectrum, Figure 3a, shows the Ni 2p_3/2_ feature at 852.7 eV with a spin‐orbit splitting of ~17.3 eV, this indicates that Ni is predominantly in metallic form, since for Ni containing oxides the spin‐orbit splitting is 17.9 eV.^[21]^ The Ni 2p region was fitted with peaks assigned to Ni^0+^ (852.7 eV/869.9 eV),^[22]^ with minor contributions of NiO (853.7 eV/871.6 eV),^[23]^ and Ni(OH)_2_ (855.9 eV/873.7 eV).^[24]^ The Ni‐oxides to Ni^0^ ratio was estimated from the fitted areas in the Ni 2p peak, resulting in a ratio of 1.41. Two additional satellites were seen in Ni 2p_3/2_ region, one corresponding to metallic Ni which is located at 6 eV away from Ni^0+^ (858.7 eV),^[22]^ and the second which appears upon NiO ionization at nearly 8 eV from NiO (861.8 eV).^[22]^ The Mo 3d region (Figure 3b) was fitted by maintaining a spin‐orbital split distance of 3.15 eV between 3d_5/2_ and 3d_3/2_.^[25]^ The latter results in the appearance of Mo^0+^ (228.0 eV/331.15 eV) as well as its oxides such as Mo^δ+^ (0<δ<4) (229.1 eV/232.25 eV), Mo^4+^ (230.3 eV/233.45 eV), Mo^5+^ (232.4 and 235.55 eV) and Mo^6+^ (234.4 and 237.55 eV).[^25^, ^26^] Note that the contribution of Mo^6+^ is much lower than Mo^5+^ (Mo^5+^/Mo^6+^=12.0) and Mo^4+^ (Mo^4+^/Mo^6+^=7.4), indicating the formation of an amorphous thin layer of MoO_3−x_ with oxygen vacancies, a typically oxide formation observed in Ni_4_Mo alloys.^[7a]^ The O 1s region, Figure 3c, depicted peaks at 530.3 eV, 531.2 eV, and 532.5 eV attributed to oxygen lattice (M−O), hydroxides (M−OH), and water adsorbed species, respectively.[^7^, ^27^] From these results, and the fact that the XPS survey spectrum reveals a higher content of Mo and O when compared to EDX analysis (Table S2), we can infer that MoO_2_ might be preferentially located at the surface.

### Catalytic Activity

**Figure 4:**
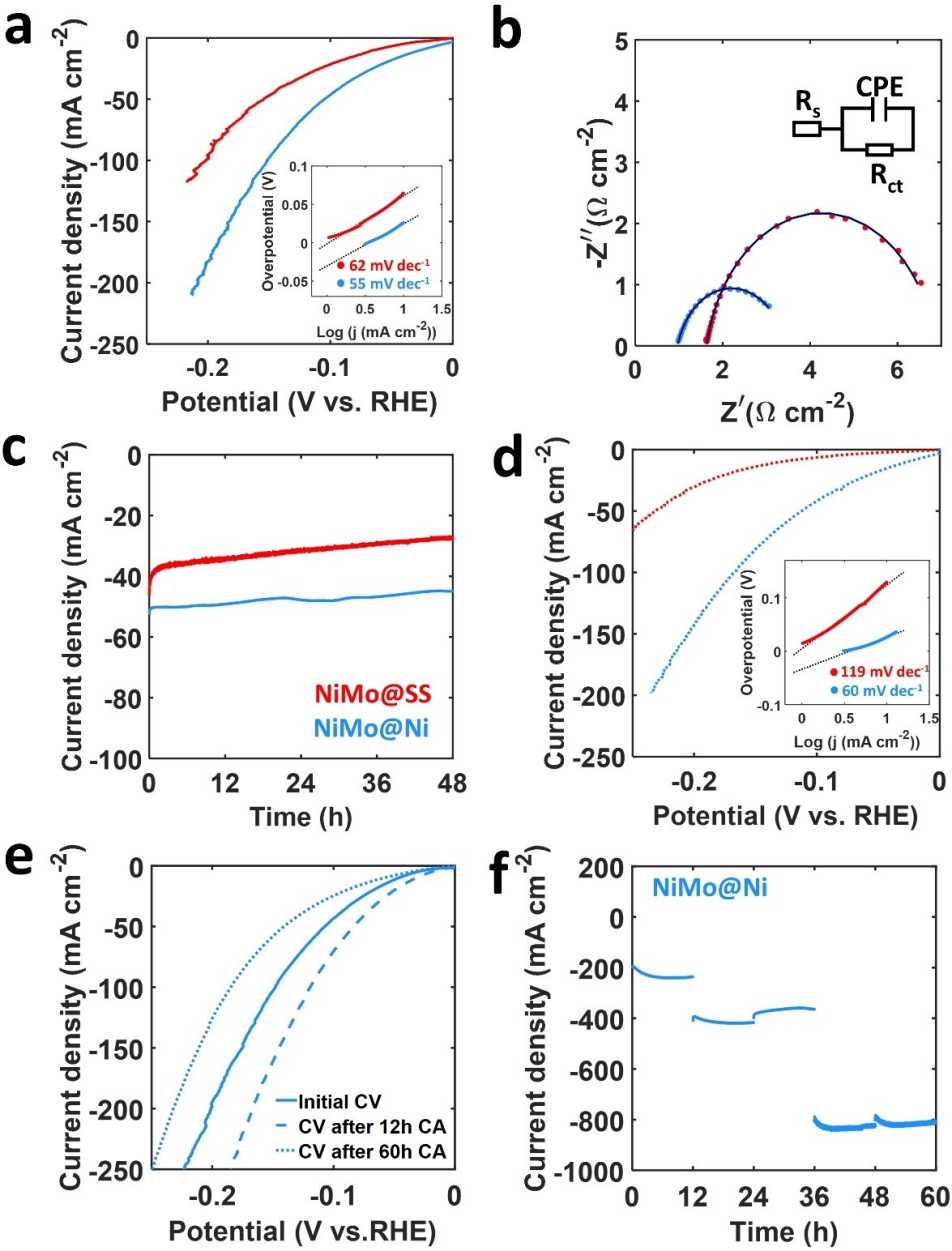
(a) iR‐corrected polarization curves and Tafel slopes, and (b) Nyquist plots and equivalent circuit of NiMo@SS and NiMo@Ni before the stability test. (c) Chronoamperometry test of both samples at a constant potential to achieve −50 mA cm^−2^ for 48 h. (d) iR‐corrected polarization curves and Tafel slopes after the stability test. (e‐f) iR‐corrected polarization curves and high‐current CA test of a different NiMo@Ni sample.

The catalytic activity towards the HER of the NiMo electrode was evaluated on two different substrates: (i) SS‐mesh, and (ii) Ni‐mesh. The HER activity was measured in a three‐electrode cell filled with 1 M Ar‐saturated KOH, see details in the experimental section. Under the selected potential window, the contribution from the SS‐mesh and Ni‐mesh are negligible, see Figure S8. The iR‐corrected polarization curves and Tafel slopes of NiMo@SS and NiMo@Ni are shown in Figure 4a. The NiMo@SS exhibit an overpotential to reach a current density of −10 mA cm^−2^ (η_10_) of 65 mV with a corresponding Tafel slope of 62 mV dec^−1^. Meanwhile, NiMo@Ni has a η_10_ equal to 26 mV and Tafel slope of 55 mV dec^−1^. This performance is in line with other Ni_4_Mo‐based electrocatalysts with a η_10_ in the range of 15 to 61 mV and a Tafel slope of 30 to 99 mV dec^−1^.[^7^, ^8^, ^28^] In our case, the values for the Tafel slope indicate that for both electrodes the HER occurs through a combined Volmer–Heyrovsky pathway,^[29]^ where the rate determining step is the electrochemical water dissociation reaction (Heyrovsky step). Electrochemical impedance spectroscopy (EIS, Figure 4b) reveals that NiMo@Ni exhibit smaller ohmic resistance (R_s_=0.97 Ω) and charge transfer resistance (R_ct_=2.5 Ω) when compared to NiMo@SS (R_s_=1.6 Ω; R_ct_=5.2 Ω). The latter strongly suggest a substrate‐dependent change in the coating that benefit the reaction kinetics when using Ni‐substrate. The electrochemical surface area (ECSA) was determined by evaluating the double‐layer capacitance, **Figure** **S9**, resulting in an area of 1447 (57.9 mF cm^−2^) and 1102 cm^2^ per cm^2^_geo_ (44.1 mF cm^−2^) for NiMo@Ni and NiMo@SS, respectively. The specific activity (Figure S10) clearly indicates a superior catalytic activity of NiMo@Ni. This result is in good agreement with the polarization curves presented in Figure 4a, where both the reaction onset potential and overpotential at −10 mA cm^−2^ are lower for NiMo@Ni when compared to NiMo@SS. Thus, the activation of the NiMo layer on Ni‐mesh requires less energy than in the case of stainless‐steel current collector due to the higher electrical conductivity of the first one, while the lower R_ct_ (related to a better electron transfer kinetics at the interface) could be caused by both an increase in surface area and a reduction in particle size, increasing the density of the electroactive sites available for the HER reaction. With all these, it is evident that Ni‐mesh is the preferred substrate.

The operational stability test was evaluated by performing 5000 CVs (0.05 V to −0.15 V vs. RHE, Figure S11) followed by a chronoamperometry test (CA) for 48 h at a constant potential required to reach −50 mA cm^−2^ (totalling ~60 h), depicted in Figure 4c–d. NiMo@SS and NiMo@Ni experienced a reduction in the current response of 41 % and 13 %, respectively. A subsequent polarisation curve, Figure 4d, reveals that NiMo@SS has an increase of 100 % in η_10_ (from 65 to 130 mV), and 50 % in η_50_ (154 to 231 mV), while the Tafel slope increases up to 119 mV dec^−1^. On the other hand, NiMo@Ni exhibits a significantly greater stability with no change in η_10_, and an increase in η_50_ of only 7 mV (105 to 112 mV), while the Tafel slope only changes to 60 mV dec^−1^. The severe degradation seen in NiMo@SS could be due to an increased corrosion rate that SS experience in alkaline environments,^[30]^ where the corrosion products at the NiMo/SS interface might compromise the adhesion and electrical contact between the active layer and the current collector. The latter further confirm the suitability of Ni‐mesh as the catalyst substrate, owing its high stability in the working solution.

We performed elemental analysis of both NiMo electrodes before and after the catalytic stress tests to investigate the corrosion of Mo, see **Table** **S1**. The results reveal that the Mo is dissolved during the HER, in agreement with previous studies.[^10^, ^14^] For NiMo@Ni, the content of Mo was reduced from 31.8 at.% (Ni : Mo=5 : 2.3) to 24.3 at.% (Ni : Mo=5 : 1.6). The latter indicates a loss of 31 % of the total Mo. A more severe Mo dissolution is observed for NiMo@SS with a reduction in Mo content of 71 %. The loss of Mo correlates well with the reduction in HER activity, where NiMo@Ni retained better HER activity than NiMo@SS.

**Figure 5:**
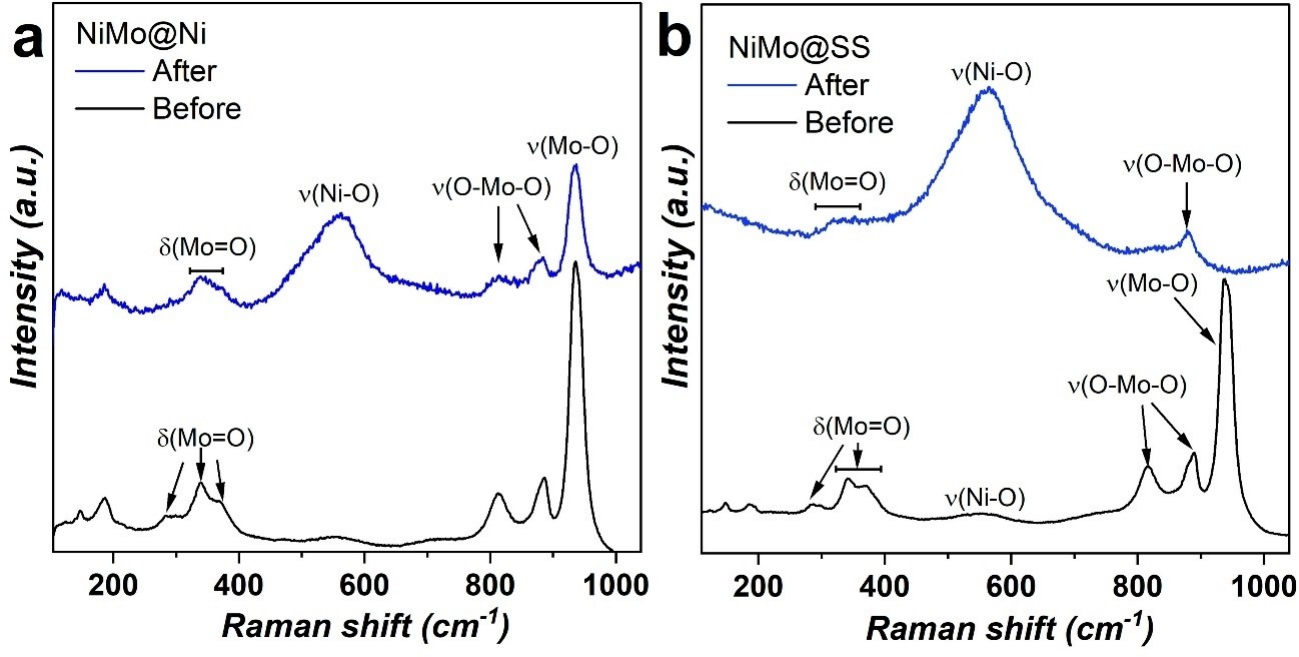
Raman spectra of (a) NiMo@Ni and (b) NiMo@SS before and after the stability test (5000 CVs (0.05 V to −0.15 V vs. RHE) followed by for 48 h at a constant potential to reach −50 mA cm^−2^.

Mo corrosion in NiMo@Ni and NiMo@SS was examined by Raman spectroscopy. Although Ni_4_Mo is not detectable by Raman spectroscopy, NiMoO_4_ oxides are partially form at the surface providing a fingerprint of the Ni_4_Mo alloy.^[31]^ The Raman spectra of both as‐produced NiMo@Ni and NiMo@SS (Figure 5) exhibit three distinct bands at 932, 887, and 811 cm^−1^ ascribed to Mo−O stretching vibrations,^[32]^ while features seen in the 350–400 cm^−1^ region are associated to Mo−O bending modes.^[33]^ Additionally, a broad band at 566 cm^−1^ is attributed to Ni−O stretching mode.^[34]^ These Raman features agrees with those seen in β‐NiMoO_4_ which has been observed previously in Ni_4_Mo catalysts.[^7^, ^32^] After the stability test, the Raman spectra of both samples now mostly comprised Ni−O (566 cm^−1^) bands^[35]^ with minimal contribution from Mo, in agreement with EDX studies. These results indicates that the source of Mo lost during the HER could be initially oxidize Mo located at the surface of active Ni_4_Mo, or other forms of oxidize Mo. Subsequently, Mo integrated in the Ni_4_Mo lattice must also leach out. Similar results have been obtained by the group of Bin Zhang where the main deactivation mechanism occur via Mo dissolution through the formation of $MoO_{4}^{<M->2}$ .^[14b]^

Giving the excellent performance of NiMo@Ni, we conducted an additional stability test. The test was initiated by applying a potential to achieve −200 mA cm^−2^ for 12 hours, followed by an increase in potential to reach −400 mA cm^−2^ for 24 hours, and then further raising the potential to achieve −800 mA cm^−2^ for another 24 hours, totalling 60 hours of operation as depicted in Figure 4e–f. As illustrated in Figure 4e, the activity of NiMo@Ni was increased after 12 h, as evidenced by the reduction in η_10_ from 47 to 32 mV when compared to the initial polarization curve. The latter could be a consequence of the dissolution of the least stable MoO_2_ phase seen at the surface and bulk of the coating, as indicated by XRD and the Raman characterizations, resulting in larger electroactive surface area, and thus electrode performance. Subsequently after the 60 h high‐current test, the η_10_ exhibited an increase of only 12 mV (47 to 59 mV), while η_50_ increased 34 mV (107 to 141 mV). These results indicate an excellent performance of the NiMo alloy deposited on Ni‐mesh electrodes for the HER reaction under various operational conditions, something not only related to its high activity, but also to the time and potential dependent stability of the Ni‐mesh current collector in alkaline media, highly desirable features for water electrolysers.

## Conclusions

We successfully synthesized highly active NiMo coatings on stainless‐steel (SS) and nickel (Ni) mesh current collectors using the solution precursor plasma spraying technique. The coatings obtained consisted in a porous arrangement of nanoparticles with diverse morphologies exhibiting an extensive surface area, something desired for applications in electrocatalysis. The electrochemical performance towards the HER was evaluated on different substrates revealing a significant influence of the substrate material on the electrocatalytic activity and stability. Ni mesh was found to be an excellent substrate when compared to SS‐mesh. The SS‐mesh suffered of high corrosion leading to a larger series resistance and reaction onset‐potential. On the other hand, NiMo@Ni achieved a remarkably competitive overpotential of only 26 mV to reach a current density of −10 mA cm^−2^, and a Tafel slope of 55 mV dec^−1^. The excellent operational stability of NiMo@Ni was evidenced by a minimal increase of only 7 mV in the overpotential at η_50_ after an extensive 60 hour stability test. EDX and Raman spectroscopy, in agreement with voltametric measurements, suggest that the primary mechanism for the degradation of the NiMo‐alloy coatings is the dissolution of different Mo oxides from the surface and its gradual leaching from the crystal lattice as function of time and potential applied. Despite this, the exceptional performance of NiMo@Ni position it as an exceptional cathode material for alkaline water electrolysis.

## Conflict of Interests

There are no conflicts to declare.

1

## Supporting information

As a service to our authors and readers, this journal provides supporting information supplied by the authors. Such materials are peer reviewed and may be re‐organized for online delivery, but are not copy‐edited or typeset. Technical support issues arising from supporting information (other than missing files) should be addressed to the authors.

Supporting Information

## Data Availability

The data that support the findings of this study are available from the corresponding author upon reasonable request.
