# Supplementary material for: Highly Active and Durable Nanostructured Nickel‐Molybdenum Coatings as Hydrogen Electrocatalysts via Solution Precursor Plasma Spraying
Source: ChemistryOpen. 2024 Oct 25;14(1):e202400069. doi: 10.1002/open.202400069 (PMC12128151; doi:10.1002/open.202400069)
Supplement: Supplementary file 1 — Supporting Information [file OPEN-14-e202400069-s001.pdf]

ChemistryOpen

Supporting Information

Highly Active and Durable Nanostructured Nickel-Molybdenum Coatings as Hydrogen Electrocatalysts via Solution Precursor Plasma Spraying

Xiuyu Wu, Alexis Piñeiro-García, Mouna Rafei, Alice Kuzhikandathil, Esdras J. Canto-Aguilar, and Eduardo Gracia-Espino*

Supplementary Information

Highly Active and Durable Nanostructured Nickel-Molybdenum Coatings as Hydrogen Electrocatalysts via Solution Precursor Plasma Spraying

Xiuyu Wu,[1] Alexis Piñeiro-García,[1,†] Mouna Rafei,[1] Alice Kuzhikandathil,[1] Esdras J. Canto-Aguilar,[1] and Eduardo Gracia-Espino[1,*]

[1] *Department of Physics, Umeå University, SE-901 87 Umeå, Sweden.*

[†] *Current affiliation: Departamento de Ingeniería Química, Alimentos y Ambiental, Universidad de las Américas Puebla, Sta. Catarina Mártir, Cholula, Puebla 72810, Mexico.*

*Corresponding author: Eduardo Gracia-Espino (eduardo.gracia@umu.se)

Table S1. *Elemental composition of NiMo coatings before and after the stability test.*

**Sample**	**Ni (at.%)**	**Mo (at.%)**	**Mo:Ni ratio (at.%/at.%)**	**Mo loss**
NiMo@SS (Before stability test)	73.5	26.5	0.361	-
NiMo@SS (After stability test)*	90.5	9.5	0.105	71 %
NiMo@Ni (Before stability test)	68.2	31.8	0.465	-
NiMo@Ni (After stability test)*	75.7	24.3	0.321	31 %
NiMo@Ni (Before stability test)	68.2	31.7	0.465	-
NiMo@Ni (After stability test)**	84.4	15.6	0.18	61 %

* After 5000 CVs (0.05 V to -0.15 V vs. RHE, **Figure S7**) followed by 48 h of chronoamperometry with a potential required to reach -50 mA cm^{-2}.

** An initial potential to achieve -200 mA cm^{-2} was applied for 12 h, then the potential was increased to reach -400 mA cm^{-2} for 24 h, and finally the potential increased once more to reach a current density of -800 mA cm^{-2} for 24 h (totalling 60 h).

Table S2. *Elemental composition from the XPS survey spectrum and EDX mapping of NiMo@SS.*

	Ni (At %)	Mo (At %)	O (At %)
XPS	21.1	13.8	65.1
EDX	57.8	19.7	22.5

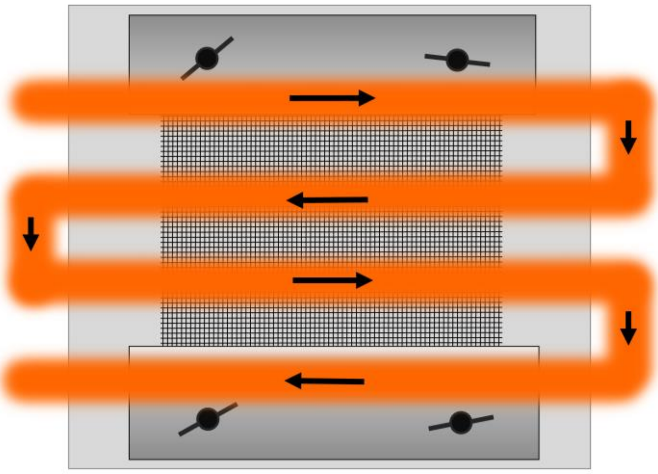

Figure S1. *Raster scan pattern used during the spraying process.*

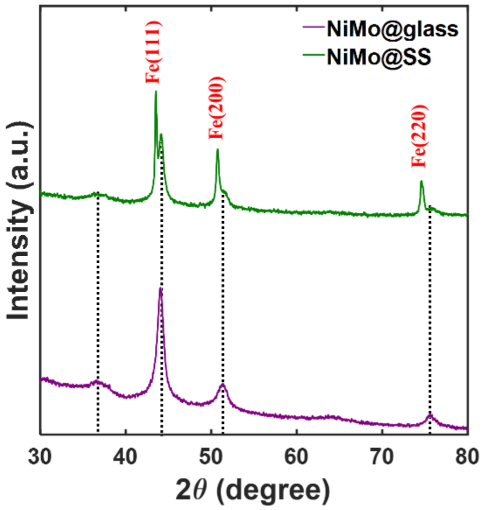

Figure S2. *X-ray diffractograms of NiMo@SS and NiMo@glass. The dash lines indicate features corresponding to the NiMo coating, which are present in both substrates. The contribution from the SS-mesh is seen as Fe with fcc crystal structure.*

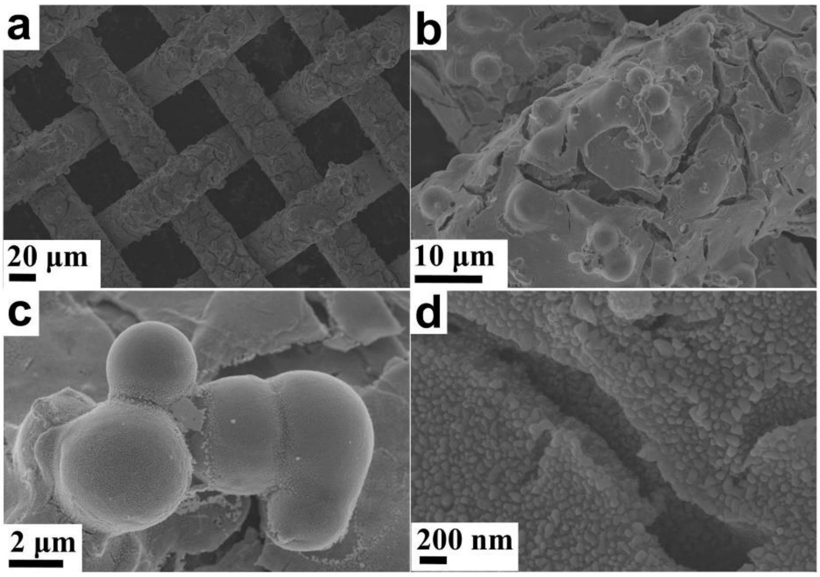

Figure S3. *(a-e) SEM images of NiMo@SS.*

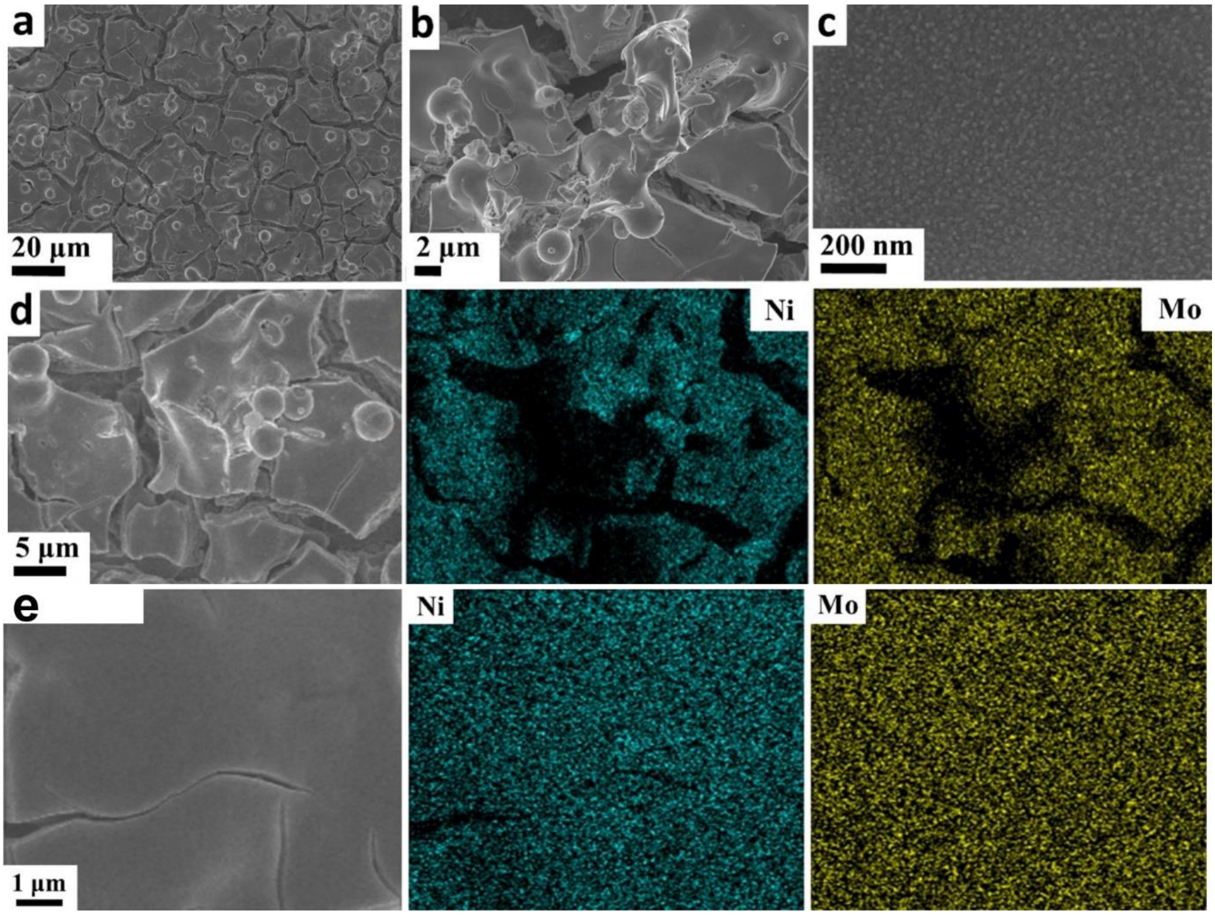

Figure S4. *(a-c) SEM images and (d-e) EDX elemental mapping of NiMo@Ni at different locations.*

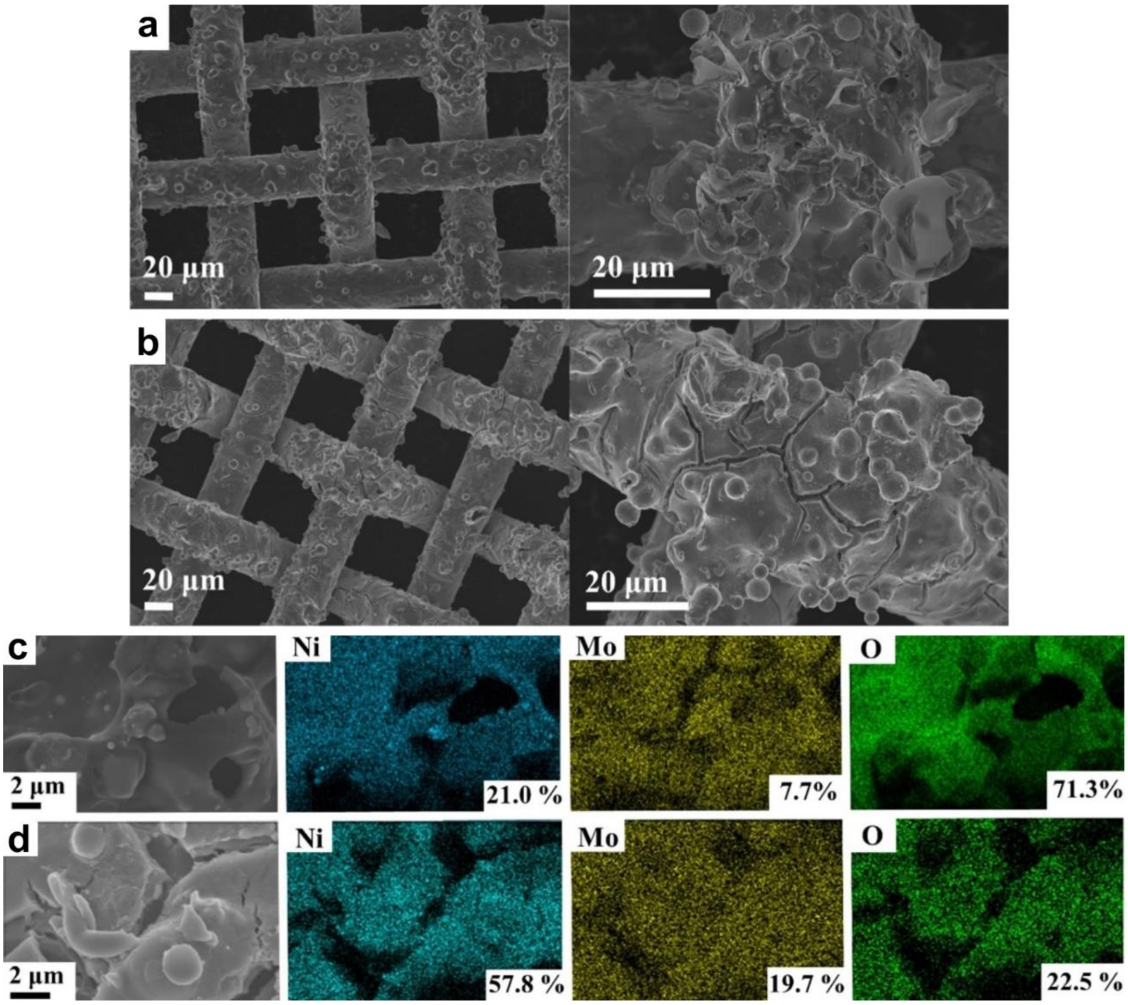

Figure S5. *Comparison of the coating before and after the thermal annealing. (a) SEM images of as-sprayed NiMo coating (before thermal annealing). (b) NiMo coating after thermal annealing (same as the images in* ***Figure S3****). (c-d) EDX elemental mapping of NiMo@SS (c) before and (d) after the annealing process.*

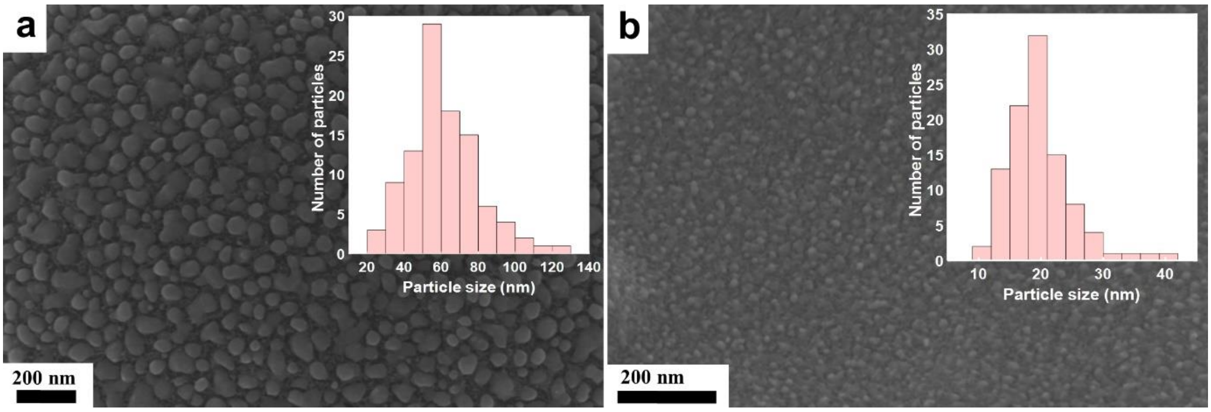

Figure S6. *Particle size distribution estimated from 100 measurement for (a) NiMo@SS and (b) NiMo@Ni.*

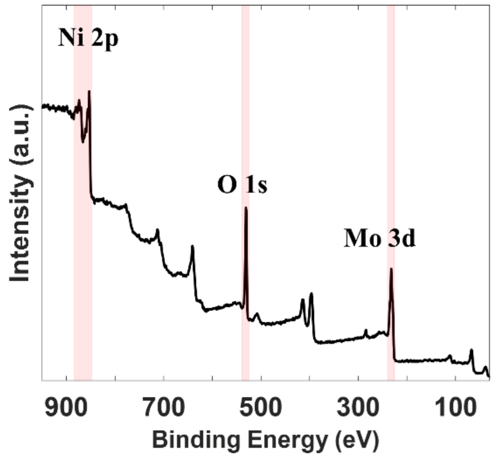

Figure S7. *XPS survey spectrum of NiMo@SS.*

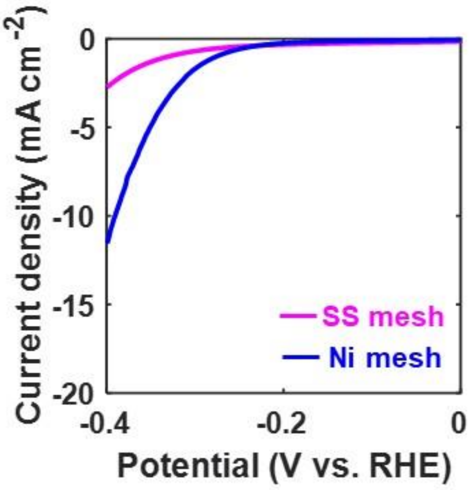

Figure S8. *Polarization curves of SS-mesh and Ni-mesh without any coating.*

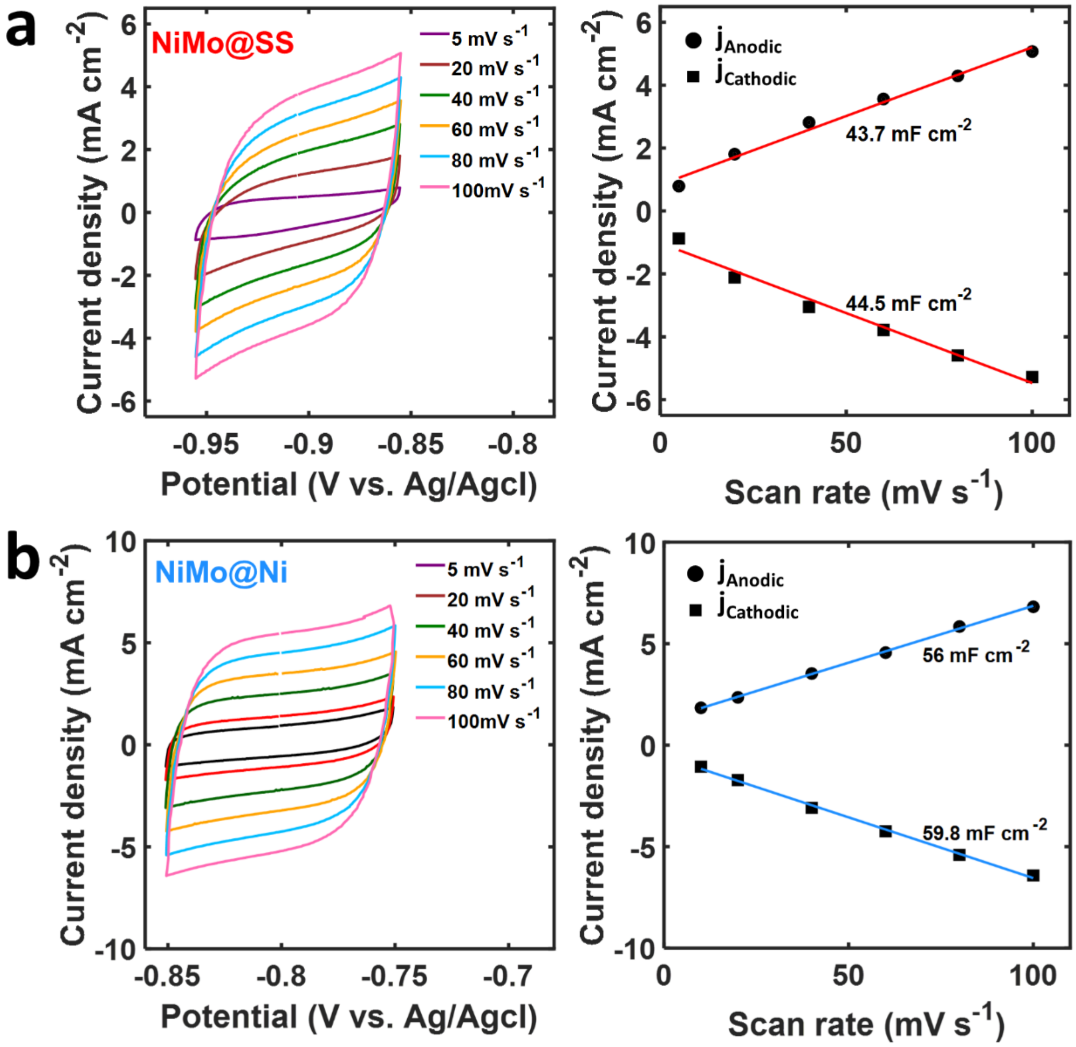

Figure S9. *Double-layer capacitance (C_{dl}) measurements of (a)NiMo@SS and (b)NiMo@Ni, the average value of C_{dl} is 44.1 mF cm^{-2} for NiMo@SS and 57.9 mF cm^{-2} for NiMo@Ni. The electrochemical surface area (ECSA) was subsequently calculated using the formula ECSA = C_{dl}/40 µF cm^{-2}, where 40 µF cm^{-2} represents the charge density for a flat surface. The calculated ECSA values are ECSA$_{NiMo@SS}$ = 1102.5 cm^2 per cm$^2_{geo}$ and ECSA$_{NiMo@Ni}$ = 1447.5 cm^2 per cm$^2_{geo.}$*

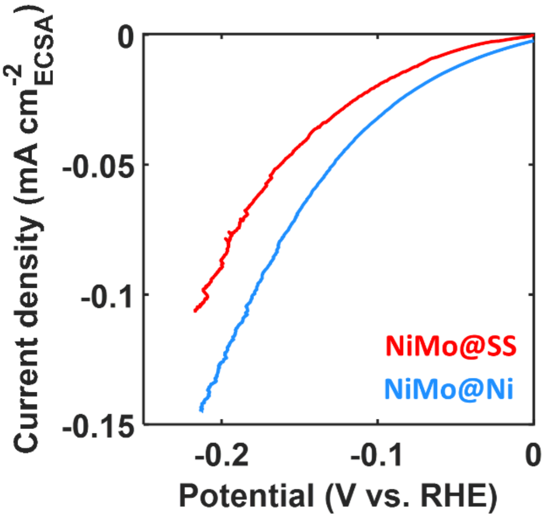

Figure S10. *Specific activity of NiMo@SS and NiMo@Ni.*

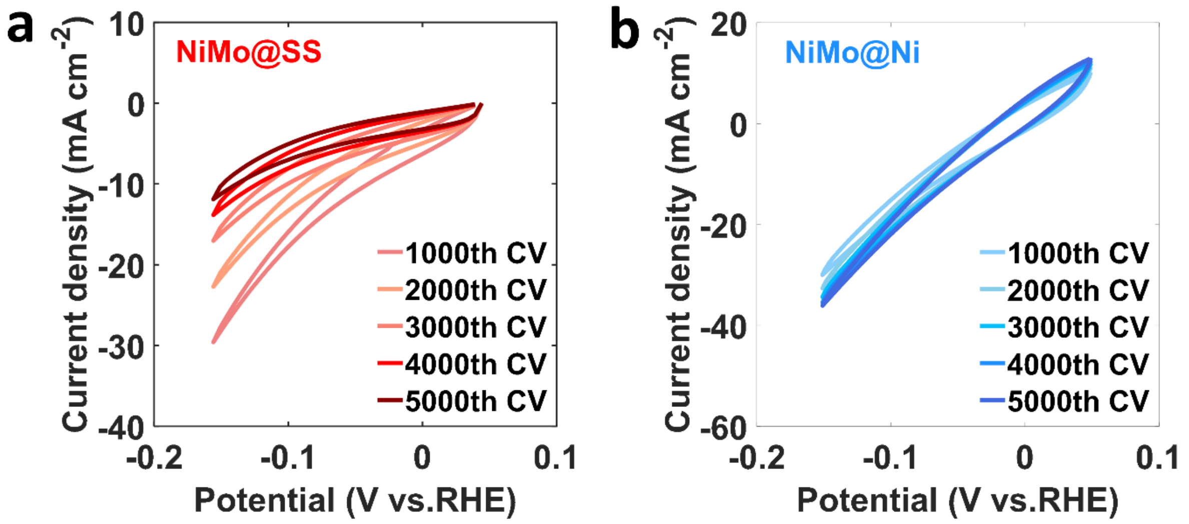

Figure S11. *Cyclic voltammetry test consisting of 5000 cycles from 0.05 to -0.15 V vs RHE. The plots show one cycle every 1000 CVs. (a) NiMo@SS, (b) NiMo@Ni.*
